# Zebrafish Brain Ventricle Injection

**DOI:** 10.3791/1218

**Published:** 2009-04-06

**Authors:** Jennifer H. Gutzman, Hazel Sive

**Affiliations:** Whitehead Institute for Biochemical Research; MIT - Massachusetts Institute of Technology

## Abstract

Proper brain ventricle formation during embryonic brain development is required for normal brain function.  Brain ventricles are the highly conserved cavities within the brain that are filled with cerebrospinal fluid.  In zebrafish, after neural tube formation, the neuroepithelium undergoes a series of constrictions and folds while it fills with fluid resulting in brain ventricle formation.  In order to understand the process of ventricle formation, and the neuroepithelial shape changes that occur at the same time, we needed a way to visualize the ventricle space in comparison to the brain tissue.  However, the nature of transparent zebrafish embryos makes it difficult to differentiate the tissue from the ventricle space.  Therefore, we developed a brain ventricle injection technique where the ventricle space is filled with a fluorescent dye and imaged by brightfield and fluorescent microscopy.  The brightfield and the fluorescent images are then processed and superimposed in Photoshop.  This technique allows for visualization of the ventricle space with the fluorescent dye, in comparison to the shape of the neuroepithelium in the brightfield image.  Brain ventricle injection in zebrafish can be employed from 18 hours post fertilization through early larval stages.  We have used this technique extensively in our studies of brain ventricle formation and morphogenesis as well as in characterizing brain morphogenesis mutants (1-3).

**Figure:**
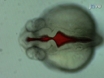


## Protocol

### Protocol

#### Part 1: Preparing for microinjection

1. Prepare for the injection by pulling capillary needles using Sutter Instruments needle puller.
2. Fill the needle with a fluorescent dye (such as Texas-Red Dextran).
3. Mount the needle in a micromanipulator and microinjection apparatus.
4. Cut the needle to the appropriate size at an angle to create a beveled tip.
5. Measure the drop size and check that it is between 1-2nl per injection in oil.

#### Part 2: Preparing the embryos

1. Inject the embryos 30 minutes earlier than the stage of interest. For example, to study the ventricles at 24 hours post fertilization (hpf), inject the embryos at 23.5 hpf. Stage the embryos according to Kimmel et al. (4).
2. Under the stereomicroscope, carefully remove the chorion from the embryos using forceps.
3. Using a plastic dish coated with 1% agarose, poke holes in the agarose with a plastic 1-200 μl pipette tip.  Carefully remove the agarose plugs from the holes using forceps.
4. Transfer the embryos in embryo media into the agarose dish with holes.
5. Add tricaine (made according to Westerfield (5)) to the media to anesthetize the embryos and prevent movement during the experiment.
6. Gently place the embryo tail down into the hole, and orient it so that the injection apparatus is on the posterior side of the embryo.

#### Part 3: Injecting the brain ventricle

1. To inject the brain ventricle, arrange the micromanipulation setup so that the needle tip is in the same field of view as the embryo on high power.
2. Carefully put the needle through the thin roofplate of the hindbrain just posterior to the r0/r1 hingepoint without hitting the brain tissue below.
3. Inject just enough dye to completely fill the ventricles. It may take several injections to fill the ventricle space, depending on the stage of the embryo.

#### Part 4: Imaging

1. Using a clean 1% agarose-coated dish, poke new holes in the dish, remove the plugs, and add tricaine to anesthetize the embryos and prevent movement.
2. Place the tail of the ventricle-injected embryo into the hole and position for a dorsal image.
3. Take an image using transmitted light.
4. It is very critical to NOT MOVE THE EMBRYO OR MICROSCOPE.
5. Change the settings on the microscope and take an image with fluorescent light.
6. Reposition the embryo to take lateral images with both transmitted light and fluorescent light.
7. Save the images as TIFF files for image processing in Photoshop.

#### Part 5:  Image Processing

1. To process the images in Photoshop, open the transmitted light and fluorescent light images of the same embryo taken in the same position.  Be sure all of the settings are the same for each image.
2. Drag your fluorescent image onto the transmitted light image and line them up exactly.
3. With the fluorescent image selected, select “Image,” “Adjustments,” and then “Replace color” and change the black background to white.
4. In the “Replace color” window adjust the “fuzziness” factor to the right until the fluorescent images looks uniform in color.
5. In the “Layers” window, select “Multiply” to view the overlay images.  Your ventricle space image should match the transmitted light image exactly.
6. Save this file and repeat for any other images.

### Representative Results/Outcome

Proper ventricle injection images are shown in Figure 1 panels A-D, while an example of incorrect injection images are shown in E-H.  Proper injections have sharp edges and non-diffuse dye.  Incorrect injection, which occurs when the needle is inserted too far into the ventricle and hits the brain tissue below, can result in dye that is visible outside of the ventricle space and in the yolk.


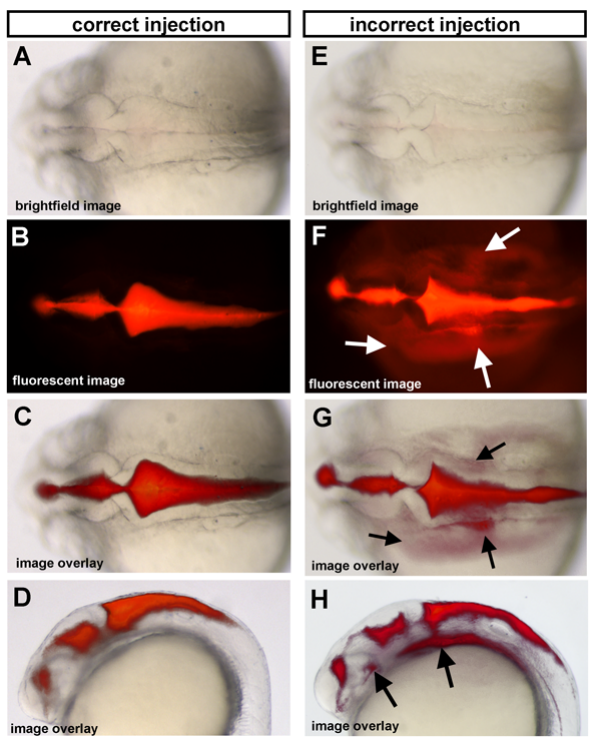


**Figure 1.  Ventricle injections of 25 hpf wild type embryos.** (A-D) Correct injection method and (E-H) incorrect injection method are shown as examples.  (A,E) Dorsal brightfield images with corresponding fluorescent images (B,F) and the overlay of the fluorescent and brightfield images (C,G) for both correct and incorrect injection methods shown.  Arrows indicate regions where dye has gone beyond the brain ventricle space due to an incorrect ventricle injection (see “Troubleshooting”).  Anterior is to the left in all images.

### Troubleshooting:

**Table:** 

Problem	Cause	Remedy
You are squashing the embryo with the needle.	Your needle is too blunt or the tip is too large.	Start with a new needle and do not break it as far back. Bevel your needle to make it sharp like a syringe needle.
The dye is throughout the embryo and the yolk (see Fig 1E-H).	Your needle went through the tissue and didn’t stay in the ventricle space.	Your needle may not be sharp enough, so you are unable to control the speed of puncture.
The dye is too faint or not in the forebrain.	You did not use a high enough pressure for the injection, or your needle was not far enough anterior to reach the forebrain when you injected the dye.	Increase your injection pressure or place your needle slightly more anterior during the injection. You can put the needle through the midbrain-hindbrain boundary if more detail is required in the forebrain. You can also simply wait a bit for the dye to further diffuse before imaging.
Your fluorescent images are dark around the edges after the color replacement in Photoshop.	You forgot to change the “fuzziness” setting in the color replace window.	Move the “fuzziness” all the way to the right in the color replace window.
The needle will not puncture the hindbrain roofplate.	Your needle is not cut properly.	Make a new needle. If you are doing a lot of injections you may need several needles as they can get dull.

## Discussion

In this video we demonstrate how to inject fluorescent dye (Texas-Red Dextran) into developing zebrafish brain ventricles.  This method is used to visualize the brain ventricle space in contrast to the surrounding neuroepithelium and is extremely useful for determining the shape of the ventricle space as well as the shape of the surrounding brain tissue.  It allows us to better understand the process of brain ventricle formation and brain morphogenesis over time in the live embryo. This technique is an excellent tool for studying brain ventricle formation defects and for initial characterization and study of brain morphogenesis mutants.
